# Interactions between Ionic Cellulose Derivatives Recycled from Textile Wastes and Surfactants: Interfacial, Aggregation and Wettability Studies

**DOI:** 10.3390/molecules28083454

**Published:** 2023-04-13

**Authors:** Catarina Costa, André Viana, Isabel S. Oliveira, Eduardo F. Marques

**Affiliations:** 1 CIQUP, IMS (Institute for Molecular Sciences), Departamento de Química e Bioquímica, Faculdade de Ciências, Universidade do Porto, Rua do Campo Alegre s/n, 4169-007 Porto, Portugal; cdcosta@centi.pt (C.C.);; 2 CeNTI—Centre for Nanotechnology and Smart Materials, Rua Fernando Mesquita, 4760-034 Vila Nova de Famalicão, Portugal

**Keywords:** sodium carboxymethylcellulose (NaCMC), quaternized cellulose (QC), ionic and nonionic surfactants, critical association concentration, contact angle

## Abstract

Interactions between polymers (P) and surfactants (S) in aqueous solution lead to interfacial and aggregation phenomena that are not only of great interest in physical chemistry but also important for many industrial applications, such as the development of detergents and fabric softeners. Here, we synthesized two ionic derivatives—sodium carboxymethylcellulose (NaCMC) and quaternized cellulose (QC)—from cellulose recycled from textile wastes and then explored the interactions of these polymers with assorted surfactants—cationic (CTAB, gemini), anionic (SDS, SDBS) and nonionic (TX-100)—commonly used in the textile industry. We obtained surface tension curves of the P/S mixtures by fixing the polymer concentration and then increasing the surfactant concentration. In mixtures where polymer and surfactant are oppositely charged (P^−^/S^+^ and P^+^/S^−^), a strong association is observed, and from the surface tension curves, we determined the critical aggregation concentration (*cac*) and critical micelle concentration in the presence of polymer (*cmc*^p^). For mixtures of similar charge (P^+^/S^+^ and P^−^/S^−^), virtually no interactions are observed, with the notable exception of the QC/CTAB system, which is much more surface active than the neat CTAB. We further investigated the effect of oppositely charged P/S mixtures on hydrophilicity by measuring the contact angles of aqueous droplets on a hydrophobic textile substrate. Significantly, both P^−^/S^+^ and P^+^/S^−^ systems greatly enhance the hydrophilicity of the substrate at much lower surfactant concentrations than the surfactant alone (in particular in the QC/SDBS and QC/SDS systems).

## 1. Introduction

Aqueous mixtures of polymers and surfactants (P/S mixtures) are colloidal systems where a number of important phase behavior and aggregation phenomena take place, among them association or segregation, coacervation or precipitation, and formation of polymer-surfactant extended complexes or gel-like networks [1,2,3,4,5,6,7,8,9,10]. Different types of interactions between polymers and surfactants (mostly hydrophobic and electrostatic, but also H-bonding, dispersive, etc.) and entropic effects (e.g., polymer chain configuration or counterion release/condensation in charged systems) lie at the root of this diverse behavior. From a fundamental point of view, we can subdivide P/S mixtures into (i) mixtures of polymers and surfactants of opposite charge (P^+^/S^−^ and P^−^/S^+^); (ii) mixtures of neutral polymers with ionic surfactants (P^0^/S^+^ and P^0^/S^−^); (iii) mixtures of similarly charged polymers (P^+^/S^+^ and P^−^/S^−^); and (iv) mixture of nonionic co-solutes (P^0^/S^0^). The most interesting mixtures are those leading cumulatively to associative phase behavior, increased interfacial activity and mixed aggregation, and they are almost invariably of type (i) and (ii) [1,3,11]. In such mixtures, the polymer chains in the bulk usually favor the aggregation of surfactant unimers yielding extended P/S complexes (“pearl necklace” micelles) at a critical association concentration (*cac*) that is much lower than the critical micelle concentration of the neat surfactant (*cmc*) [1,3,11]. Moreover, in the P/S system, the surfactant forms individual (polymer-free) micelles at a *cmc*^p^ that, in turn, is higher than *cmc*, depending on P concentration. In P^0^/S^+^ and P^0^/S^−^ mixtures, both hydrophobic (predominantly) and electrostatic interactions play a role in the association. Due to the strong electrostatic interactions at play, mixtures of oppositely charged solutes (P^+^/S^−^ and P^−^/S^+^) also tend to form insoluble complexes, showing phase separation either in the form of coacervation (liquid-liquid separation) or precipitation (solid-liquid separation) [1,2,6]. Typically phase separation occurs at or in the vicinity of charge neutrality, but redissolution takes place at a sufficiently high excess charge of either polymer or surfactant.

Besides their relevance to fundamental colloidal studies, P/S systems have also been addressed as models to study lipid/protein interactions in biological membranes [5,12,13,14,15] and exploited for numerous applications such as microencapsulation and emulsification [16], fabric softening [17], viscosity modulation of colloidal suspensions [18,19], drug delivery [20,21], dispersion of carbon nanomaterials [22] and tissue regeneration [23]. Owing to their importance for all these applications, mixtures of water-soluble polymers and common surfactants have been subject to numerous studies [24,25,26,27,28].

Cellulose is a polymer that does not dissolve in water, yet it can be chemically modified to become water-soluble. Studies on the interactions of water-soluble cellulose derivatives and surfactants have attracted great interest [6,17,29,30,31,32,33,34,35,36]. These particular P/S systems are relevant for technical applications, like the formulation of detergents, fabric softeners and paints, and tertiary oil recovery, among others [26,36,37,38]. Given that in this work we prepared and studied water-soluble cellulose derivatives obtained from textile wastes, we shall focus on the importance of P/S mixtures for textile applications. Apart from lowering the surface tension of aqueous solutions, surfactants act as antistatic, untangling and softening agents for textiles [37,38,39]. The choice of a particular surfactant for a particular purpose depends on its ability to interact with the fibers and/or other components of textiles [39]. Fabric softeners are water-based agents used in the rinse cycle to impart softness to cotton fabrics (cellulose) and provide soft handling and smoothness to clothes. They are usually formulated not only with surfactants but polymers, the role of which is to adjust the surfactant content and deposition behavior, and the softening and fragrance delivery performances [38]. The softening mechanism is mainly attributed to the prevention of a crosslinked network by water [40], yet fabric softeners often impair the hydrophilicity of fabrics. Hence, it becomes important to obtain solutions that promote softness without being detrimental to hydrophilicity. The use of cellulosic polymers as textile finishing agents is an area that is still largely unexplored. Usually, the modification is carried out directly on the cotton fiber, but with increasing environmental awareness and demand for greener finishing alternatives, cellulosic polymers are expected to be increasingly employed as finishing agents. Since, in many of these instances, they will be mixed with surfactants, it is critical to fundamentally understand the solution behavior of P/S systems containing cellulose derivatives.

**Figure 1:**
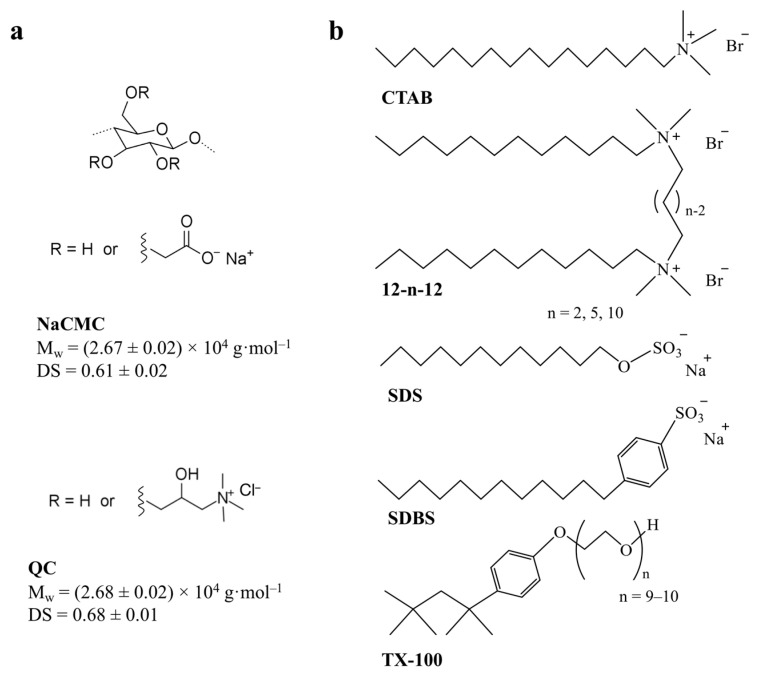
Molecular structure of: (**a**) cellulosic polymers, NaCMC (sodium carboxymethylcellulose) and QC (quaternized cellulose); and (**b**) surfactants used in this work, CTAB (cetyltrimethylammonium bromide), 12-n-12 (bis-quat gemini surfactants), SDS (sodium dodecylsulfate), SDBS (sodium dodecylbenzenesulfonate) and TX-100 (Triton X-100).

Here, our aim was to study P/S interactions using sodium carboxymethylcellulose (NaCMC) and a cationic quaternized cellulose (QC) derivatized from cellulose recycled from textile wastes (Figure 1a). Due to the synthetic process, these polymers have a lower degree of polymerization (DP) than their commercial counterparts [41]. On the other hand, by using polymers obtained from textile recycling, a circular economy concept and better management of natural resources are promoted. Carboxymethylcellulose (an anionic derivative, P^−^) is the most industrially applicable cellulose ether, finding numerous applications in the food, pharmaceutical, cosmetic, and textile industries [42]. Its properties—namely viscosity, solubility and biodegradability—are highly dependent on the resulting degree of substitution (DS), where the theoretical maximum is 3. It is soluble in water for DS > 0.4, and its biodegradability decreases with increasing DS. In the literature, a few reports can be found on the interactions of various commercial NaCMCs with cationic surfactants, where complex formation was analyzed [31,32,43]. Cationic cellulose derivatives, like QC (P^+^), have several interesting characteristics, such as hydrophilicity, biodegradability and antibacterial properties, and as such, are used in diverse applications, particularly in the textile, paper, food, cosmetics, chemical and pharmaceutical industries [44]. The interactions of various types of commercial cationic celluloses (namely derivatives of hydroxyethyl cellulose) with surfactants, particularly with anionic surfactants [45,46,47,48,49,50,51] (systems with direct relevance to fabric softeners, hair conditioners and paints), have been previously reported, with focus on the characterization of complex formation, interfacial and rheological properties.

In this work, we conducted an extensive study of the interfacial and aggregation interactions of the synthesized NaCMC and QC with assorted surfactants—cationic (S^+^: CTAB and gemini), anionic (S^−^: SDS and SDBS) and nonionic (S^0^: TX-100)—commonly used in the textile industry (Figure 1b). P/S systems with solutes of opposite charge (P^+^/S^−^ and P^−^/S^+^), same charge (P^+^/S^+^ and P^−^/S^−^) or with a neutral surfactant (P^+^/S^0^ and P^−^/S^0^) were studied, and their ability to change the hydrophilicity of textile substrate was also evaluated.

## 2. Results and Discussion

### 2.1. Polymer-Surfactant Interactions: Surface Tension, Zeta Potential and pH Data

Surface tension curves were obtained for the different types of P/S systems defined, consisting of systems of opposite charge (P^−^/S^+^ and P^+^/S^−^), similar charge (P^−^/S^−^ and P^+^/S^+^) and with nonionic surfactants (P^−^/S^0^ and P^+^/S^0^). To build each curve, the polymer concentration was kept fixed (either 0.01, 0.05, 0.1 or 0.5 wt%), and the surfactant concentration (*m*_s,_ in molality) varied for a few orders of magnitude. The surface tension of the individual polymer solutions was found to be virtually independent of concentration (within this range) and equal to 60 mN·m^−1^ for NaCMC and to 65 mN·m^−1^ for QC, indicating that these polymers are only weakly surface-active. In some specific points of the surface tension curves (indicated by arrows in the respective graphs), zeta potential and pH measurements were done to monitor how these parameters were affected by the P/S interactions.

**Figure 2:**
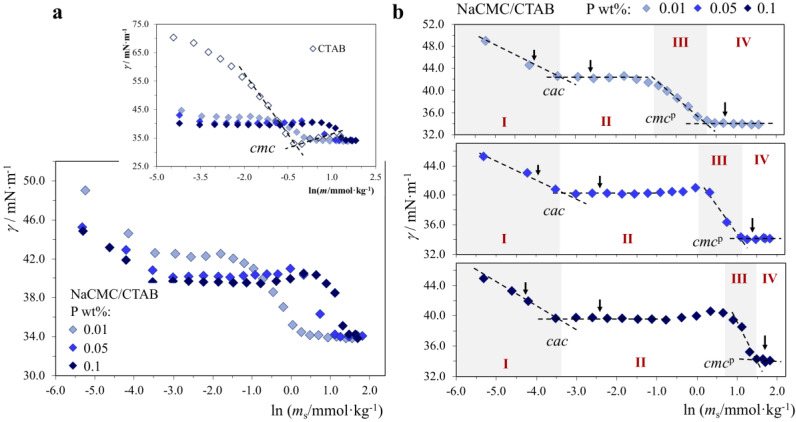
Surface tension curves for neat CTAB (30.0 °C) and NaCMC/CTAB mixtures (25.0 °C), where the polymer (P) concentration in each curve is constant and equal to 0.01, 0.05 or 0.1 wt%, and the surfactant (S) concentration is varied: (**a**) all P/S systems, inset with CTAB curve; (**b**) individual P/S curves. Legend: *cmc*, neat surfactant critical micelle concentration; *cac*, critical association concentration, *cmc*^p^, surfactant *cmc* in the presence of polymer. Arrows: zeta potential and pH measurements. Regions I–IV: different behavior regions.

**Figure 3:**
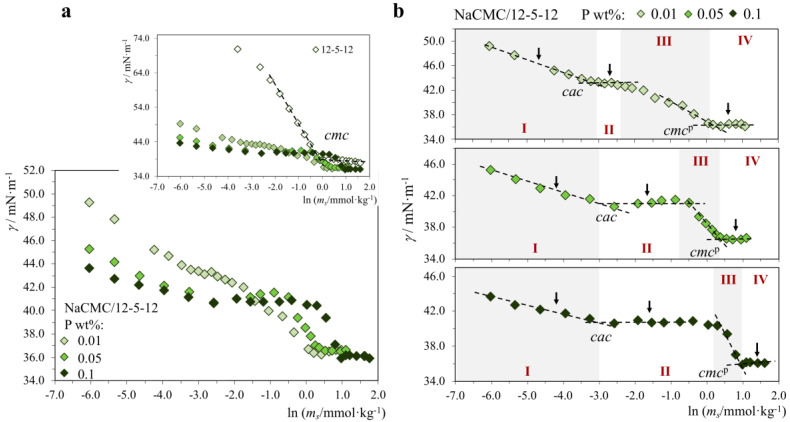
Surface tension curves for neat 12-5-12 and NaCMC/12-5-12 mixtures (25.0 °C), where the polymer (P) concentration in each curve is constant and equal to 0.01, 0.05 or 0.1 wt%, and the surfactant (S) concentration is varied: (**a**) all P/S systems, inset with 12-5-12 curve; (**b**) individual P/S curves. Legend: *cmc*, neat surfactant critical micelle concentration; *cac*, critical association concentration, *cmc*^p^, surfactant *cmc* in the presence of polymer. Arrows: zeta potential and pH measurements. Regions I–IV: different behavior regions.

**Figure 4:**
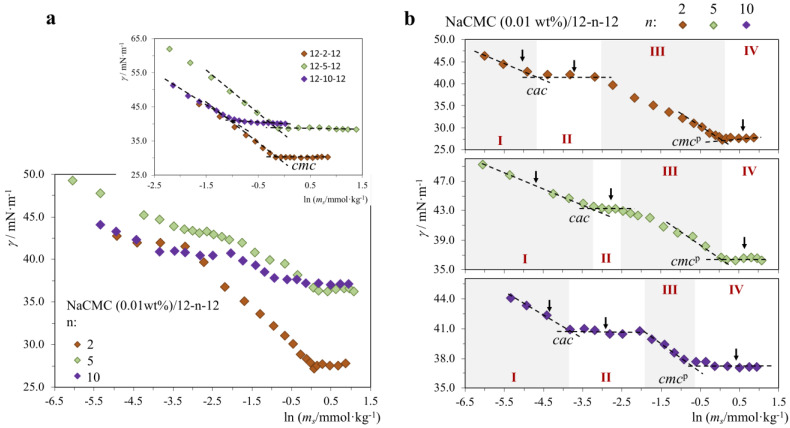
Surface tension curves for the neat 12-n-12 gemini (n = 2, 5 and 10) and NaCMC/12-n-12 mixtures (25.0 °C), where the polymer (P) concentration in each curve is constant and equal to 0.01 wt%, and the surfactant (S) concentration is varied: (**a**) all P/S systems, inset with the 12-n-12 curves; (**b**) individual P/S curves. Legend: *cmc*, neat surfactant critical micelle concentration; *cac*, critical association concentration, *cmc*^p^, surfactant *cmc* in the presence of polymer. Arrows: zeta potential and pH measurements. Regions I–IV: different behavior regions.

**Figure 5:**
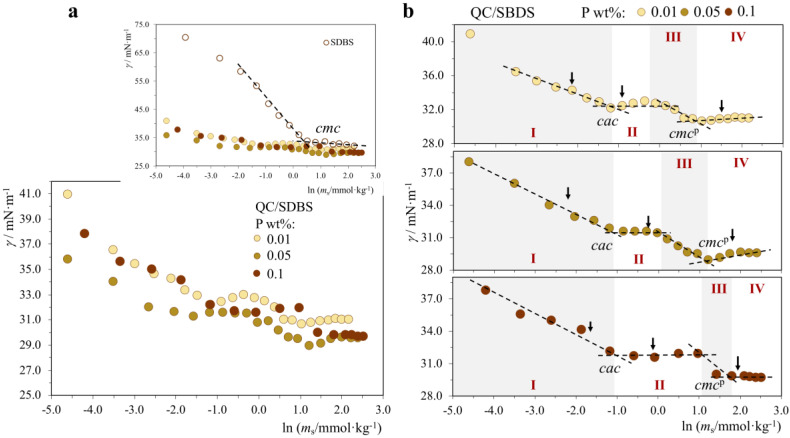
Surface tension curves for neat SDBS and QC/SDBS mixtures (25.0 °C), where the polymer (P) concentration in each curve is constant and equal to 0.01, 0.05 or 0.1 wt%, and the surfactant (S) concentration is varied: (**a**) all P/S systems, inset with SDBS curve; (**b**) individual P/S curves. Legend: *cmc*, neat surfactant critical micelle concentration; *cac*, critical association concentration, *cmc*^p^, surfactant *cmc* in the presence of polymer. Arrows: zeta potential and pH measurements. Regions I–IV: different behavior regions.

As mentioned above, P^+^/S^−^ and P^−^/S^+^ systems often display the formation of insoluble complexes and phase separation (coacervation or precipitation), namely for charge neutrality composition and its vicinity. Typically, when there is a significant excess of polymer charge or surfactant charge, the P/S system is completely soluble. For the current P^−^/S^+^ (Figure 2, Figure 3 and Figure 4) and P^+^/S^−^ systems (Figure 5), in region I of the surface tension curves, there is a high polymer/surfactant charge ratio (of the order of 10–10^3^, depending on the system), i.e., there is a significant excess of polymer charge. Conversely, in region IV, there is a high surfactant/polymer charge ratio (of the order of 2–100, depending on the system), i.e., there is an excess of surfactant charge. This obviously means that at some intermediate point along the curve, the charge neutrality point is crossed; despite this fact, no phase separation was observed for any of the systems investigated. Consequently, any P/S complexes formed in these systems under the studied conditions are water-soluble.

#### 2.1.1. P^−^/S^+^ Systems: Strong Association

*NaCMC/CTAB system*. For the NaCMC/CTAB system, the surface tension graphs obtained (Figure 2) indicate that there is a strong interaction in solution between polymer and surfactant since one observes a critical association concentration (*cac*) that is significantly lower than the neat surfactant *cmc*. One can identify 4 regions of behavior, as indicated in the individual P/S graphs in Figure 2b. In region I, the surface tension decreases gradually with increasing surfactant concentration, and what is striking is that the PS solution already presents much lower surface tension than the surfactant alone (see top graph in Figure 2a) or the polymer alone. In this region, surfactant unimers adsorb at the liquid-gas interface, and this adsorption is greatly enhanced in the presence of polymers; presumably, adsorbed P/S complexes may even form. In region II, a plateau appears. The *cac* signals the surfactant concentration where micellar-like aggregates form in the close vicinity of the polymer chains in the bulk—the “pearl necklace” PS structures [1,3,8]. The surface tension remains constant as all new surfactant binds to the bulk polymer. This goes until all polymer chains are “saturated” with surfactant. At the point signaled as *cmc*^p^, free surfactant-only micelles form in the bulk (with *cmc*^p^ higher than *cmc*). At any surfactant concentration above *cmc*^p^, the solution is composed of free CTAB unimers, mixed NaCMC/CTAB aggregates and CTAB-only micelles, the key point here being that the anionic polymer exists strongly associated with the cationic surfactant. Our results are in qualitative agreement with those previously reported on a mixture of commercial NaCMC and CTAB [43].

In Table 1, we can see that the *cac* is ≈0.04 mmol·kg^−1^ (ca. 20 times lower than the surfactant *cmc*) and basically independent of polymer concentration, at least in the range 0.01–0.1 wt% investigated; however, Figure 2b also shows that the *cac* plateau becomes bigger as the polymer concentration in the system is increased. The obvious consequence is that, contrary to *cac*, the *cmc*^p^ increases gradually as polymer concentration is increased—this comes as no surprise since more surfactant is consumed to saturate the polymer chains before individual surfactant micelles can form.

If we now look at the variation of zeta potential, *ζ*, and pH measured at some points of the curves, several interesting facts emerge (Table 1). The number of points measured in each curve (three) is limited, but our goal was to have a preliminary qualitative picture of the main trends. The polymer chains alone show *ζ* ≈ −45 mV (with virtually no P concentration effects), while individual CTAB micelles show *ζ* ≈ +42 mV (at 10 mmol·kg^−1^). Surprisingly, in region I, the *ζ* of the P/S mixture becomes slightly more negative (by about −2 to −6 mV) than the polymer alone, despite the fact that a cationic surfactant has been added. Though small, this effect is consistent in all 3 mixtures studied, and it cannot be attributed to uncertainties in *ζ*. In region II, where the P/S aggregates exist in the bulk, *ζ* remains negative, but now the absolute values decrease, as expected, due to the neutralization of the polymer chains by the added surfactant. In region IV, *ζ* is reversed to positive values (≈+34 mV), indicating that the contribution of the cationic CTAB micelles now present become dominant in the measured *ζ*. Still, *ζ* of the P/S system (≈+34 mV) is markedly below that of the neat CTAB micelles (+42 mV), an effect that can be reasonably assigned to the presence of the anionic polymer in the system. If we look at the pH variation, upon addition of CTAB, the solution goes from slightly alkaline (due to NaCMC and its basic carboxylate group) to slightly acidic (due to CTAB, which per se originates pH ≈ 5.9).

*NaCMC/gemini system*. The surface tension curves for P/S mixtures involving the bis-quat gemini 12-5-12 are shown in Figure 3. Gemini surfactants are dicationic molecules of the general type m-n-m, containing two alkyl chains (m length) and a covalent alkyl spacer (n length) linking the two charged groups (Figure 1b). Spacers typically vary between n = 2 and n = 12, so for this study, we chose an intermediate one (n = 5). It is interesting to note that despite the very different molecular architecture of the cationic surfactant, the main trends observed for the NaCMC/12-5-12 system (Table 2) are fairly similar to those of the NaCMC/CTAB above, namely: (i) regions I-IV are clearly found; (ii) the P/S mixture is extremely surface active in region I; (iii) *cac* is almost independent of polymer concentration and about 15 times smaller than the *cmc* of neat 12-5-12; (iv) *cmc*^p^ increases steadily with increased polymer concentration. Moreover, the curves obtained here are also essentially consistent with the few literature reports on the interactions of commercial NaCMC with gemini surfactants, namely with 14-4-14 [31] and 12-6-12 [32], for which soluble P/S complexes were also observed and respective *cac* values determined. Notably, the *ζ* and pH of these mixtures (Table 2) also follow similar patterns to the NaCMC/CTAB one, including the counterintuitive appearance of a more negative *ζ* of the mixture in region I when the cationic surfactant is added. In region IV, *ζ* is reversed to positive values (≈+22 mV) as expected, but it is well below the value for the neat gemini micelles (+57 mV), which indicates a more significant impact of the polymer on the charges of the system, compared to the NaCMC/CTAB. Regarding pH, the variation is weak but there is also a trend: the pH decreases as gemini is added, even though in this case the surfactant solution per se is basically neutral.

It is known that many physicochemical properties of gemini surfactants are critically dependent on the spacer length (when the main alkyl tail lengths are kept constant in a family of molecules), including the *cmc*, adsorption on surfaces, dispersion capacity and thermotropic liquid crystalline behavior [52,53,54]. Here, we explored the influence of the spacer length of the gemini surfactants on their interactions with NaCMC, and for this, we compared the behavior of 12-2-12 (short spacer), 12-5-12 (intermediate spacer) and 12-10-12 (long spacer) in the presence of 0.01 wt% polymer. The results are shown in Figure 4 and Table 3.

First, the marked effect of the influence of the spacer length on the *cmc* and surface activity of the neat gemini can be seen in the inset in Figure 4a and values in Table 3. The trend in *cmc* is 12-10-12 << 12-2-12 < 12-5-12, which shows that the most hydrophobic gemini (longest and most flexible spacer) has the lowest *cmc*, as one would expect. Yet, the order between spacers 2 and 5 seems opposite to expectation, as the shortest spacer has the lowest *cmc*. The explanation is that spacer 5 is not flexible enough to bend inwards towards the micellar core, and as a consequence, this creates an interfacial Gibbs energy penalty (higher exposure to water) that is detrimental to micellization; for spacer 2, the same penalty exists but is smaller in magnitude due to the shorter number of CH_2_ groups. In terms of surface activity (as measured by *γ_cmc_*), the trend is 12-5-12 ≈ 12-10-12 << 12-2-2, i.e., the latter is the most surface active molecule (lowest *γ_cmc_*). When we analyze the effect of the spacer on the P/S systems, we observe that with respect to the *cac,* the order is 12-2-12 < 12-10-12 < 12-5-12. This order suggests that, at lower surfactant concentrations, the gemini with the shortest and longest spacer seem to interact a bit more favorably with the polymer than 12-5-12. A hypothesis is that shorter distances between the cationic charges may lead initially to more favorable electrostatic interactions with the polymer charges (similar effects were reported before [55]), thus favoring the mixed micellization. The spacer in 12-10-12 can bend inwards and thus have charges effectively closer than 12-5-12. However, interestingly, the *cmc*^p^ follows the same pattern as the *cmc* (Table 3), 12-10-12 << 12-2-12 < 12-5-12, which suggests that when the gemini concentration is high enough, the surfactants in the presence of the polymer interact in a similar way as when they are alone in the bulk, and so the “regular” order appears. Noteworthy is that at *cmc*^p^, all P/S systems are more surface-active (lower *γ*) than the neat S systems, and the order is 12-5-12 ≈ 12-10-12 << 12-2-2, identical to that observed for the neat surfactants. Looking at the variation in *ζ,* we observe similar trends between the gemini, common to all the cationic surfactants studied here (CTAB and gemini). The pH of the NaCMC/gemini systems seems to be fairly insensitive to surfactant concentration, remaining essentially around 7.

#### 2.1.2. P^+^/S^−^ Systems: Strong Association

*QC/SDBS system.* Similar to the P^−^/S^+^ systems described above, in the QC/SDBS system, where now the polymer is cationic (P^+^) and the surfactant anionic (S^−^), there is a strong association in solution, and regions I–IV can be likewise identified (Figure 5). Again, we observe a *cac* ≈ 0.33 mmol·kg^−1^ (independent of P concentration) that is lower than the neat surfactant *cmc*; however, in this case, the decrease is only by about three times (Table 4), whereas in all P^−^/S^+^ the reduction was within 15-20 times. We can speculate that the benzene ring of SDBS may cause some steric hindrance with the polymer segments and thus make the P/S electrostatic interactions less than optimal. In any case, following the usual pattern, as the polymer concentration is increased, the *cac* plateau becomes more extended, so that *cmc*^p^ gradually increases. Noteworthy, at the *cmc*^p^ plateau, the surface tension values, *γ_cmc_*^p^ ≈ 30 mN·m^−1^, are the lowest observed in all P/S mixtures investigated in this work, indicating that this P/S system is very surface-active (the comparison with NaCMC/CTAB, *γ_cmc_*^p^ ≈ 34 mN·m^−1^, is particularly illustrative of this point). Turning our attention to the variation of *ζ* (Table 4), there is a subtle difference with respect to the previous P^−^/S^+^ systems: upon the addition of an anionic surfactant to region I, *ζ* immediately becomes less positive, as reasonably expected. Further addition of surfactant eventually reverses *ζ* to negative values (≈−40 mV) when the anionic free SDSB rich micelles are present in the solution. Still, the value is sizably less negative than that of neat SDSB micelles (−56 mV) due to the presence of the cationic polymer in the solution. Regarding pH, for 0.01 and 0.05 wt% polymer, it remains basically neutral (pH ≈ 7.0–7.5) and independent of surfactant concentration; however, the initial 0.1 wt% polymer solution is alkaline (pH = 8.4), and addition of surfactant brings it to almost neutral (pH ≈ 7.6).

*QC/SDS system.* The results for the QC/SDS system can be found in Figure S1 and Table S1. This system follows similar patterns to QC/SDBS, but there are also a few differences. The SDSB and SDS surfactants differ significantly in the fact that the former contains a benzene ring between the alkyl chain and the charged headgroup. This justifies the much smaller *cmc* of SDBS compared to SDS since the nonpolar benzene group adds to the magnitude of the hydrophobic effect driving micellization. Concerning the variation of ζ and pH (Table S1), the trends are very similar to those of the QC/SDBS system. Overall, the basic patterns of P/S behavior of two systems are fairly analogous, despite the molecular differences between the two surfactants. This seems to underpin the key role played by the intrinsic properties of the cationic polymer, QC, in its interactions with anionic surfactants. 

#### 2.1.3. P^+^/S^+^, P^−^/S^−^, P^+^/S^0^ and P^−^/S^0^ Systems: No Interactions with the Exception of QC/CTAB

Having studied the P/S systems of opposite charge, it was also relevant to evaluate whether NaCMC and QC had any type of interactions with surfactants of similar charge (P^+^/S^+^ and P^−^/S^−^ systems) and with the nonionic surfactant TX-100 (P^+^/S^0^ and P^−^/S^0^ systems). Previous studies in the literature indicated that in P/S systems of similar charge or involving nonionic surfactants, the almost universal behavior is that of no interactions between the two co-solutes, and in some cases, even segregative phase separation is observed, implying the existence of intrinsically repulsive interactions [1,2,3]. Thus, it was not surprising to observe that no co-solute interactions exist in solution in the P^−^/S^−^ systems, NaCMC/SDBS and NaCMC/SDS (Figure S2), and the P^+^/S^0^ and P^−^/S^0^ systems, NaCMC/TX-100 and QC/TX-100 (Figure S3). For all systems, the surface tension curves of the P/S are basically superimposable with that of neat surfactant, indicating that polymer and surfactant molecules virtually do not interact with each other in solution. The graphs shown are for 0.01 wt% polymer, but even at higher polymer concentrations, no changes were observed.

**Figure 6:**
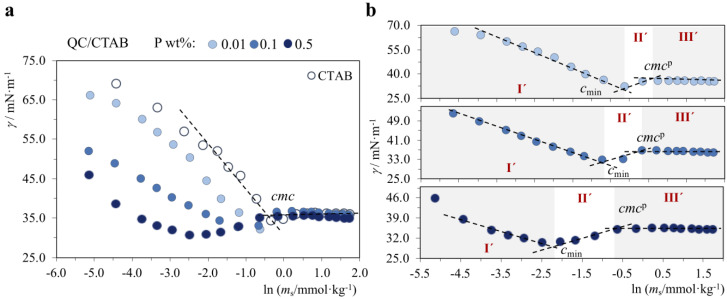
Surface tension curves for neat CTAB (30.0 °C) and QC/CTAB mixtures (25.0 °C), where the polymer (P) concentration in each curve is constant and equal to 0.01, 0.1 or 0.5 wt%, and the surfactant (S) concentration is varied: (**a**) all P/S systems; (**b**) individual P/S curves. Legend: *cmc*, neat surfactant critical micelle concentration *c*_min_, surfactant concentration at which a minimum in *γ* is observed; *cmc*^p^, surfactant *cmc* in the presence of polymer. Regions I’–III’: different behavior regions.

On the other hand, a different and unexpected behavior was observed for the P^+^/S^+^ system (QC/CTAB), where we found some form of associative interaction between P^+^ and S^+^, as demonstrated by the surface tension curves in Figure 6. Firstly, the surface tension graphs show that this P/S system is much more surface active than the surfactant alone, at the same surfactant concentration; besides, this effect is enhanced as the polymer concentration is increased from 0.01 to 0.1 and then to 0.5 wt%. Secondly, the graphs follow a similar profile, and three regions I’–III’ can be identified (Figure 6b). In region I’, the surface tension decreases continuously down to a minimum, yielding the point defined here as *c*_min_. In region II’, it goes up again, and in region III’ there is the common micellization plateau, starting at a concentration designated here as *cmc*^p^. Interestingly, we see that upon increasing polymer concentration, the extension of the plateau increases, and both *c*_min_ and *cmc*^p^ decrease (Table 5).

An explanation for the behavior of this P^+^/S^+^ mixture is not straightforward, but we will advance a hypothesis. To our knowledge, this type of behavior has not been reported before. It suggests the existence of an attractive interaction between QC and CTAB, possibly of hydrophobic nature. The markedly reduced values of surface tension in region I’, down to *c*_min_, in the P/S system compared to the neat surfactant indicate that the polymer favors the adsorption of surfactant unimers at the gas-liquid interface, suggesting that some kind of P/S complexes may even form at the surface. The subsequent increase in surface tension in region II’ could be explained if surfactant aggregates meanwhile formed in the bulk start removing the “complexes” from the surface, originating mixed QC/CTAB aggregates in the bulk. Once the removal process is finished, surfactant unimers adsorb at the surface, and CTAB-only micelles form in the bulk at *cmc*^p^ in a regular micellization process, justifying the plateau observed. Further investigations will have to be performed to see if other similarly charged systems show this type of behavior and to underpin the mechanism behind it.

### 2.2. Effect of Polymer-Surfactant Interactions on Wettability

To investigate the effect of the P/S systems on the wettability of surfaces, contact angle (*θ*) measurements were performed on P/S aqueous droplets deposited on a hydrophobic textile substrate. If *θ* ≥ 90º, the substrate remains poorly wetted by water, i.e., hydrophobic. If *θ* < 90º, it is partially wetted by water, and the lower the *θ*, the more hydrophilic it becomes. If *θ* = 0º, the substrate is totally hydrophilic. These studies are relevant for the potential development of fabric softeners with improved properties. The surface of fabrics and clothes treated with softeners usually becomes hydrophobic, and thus water cannot quickly absorb between the fibers [37,39]. Ideally, one should achieve the softening effect and preserve the hydrophilicity of the fibers. This study aimed to determine which P/S mixture (and which compositions), if any, favor substrate hydrophilicity.

**Figure 7:**
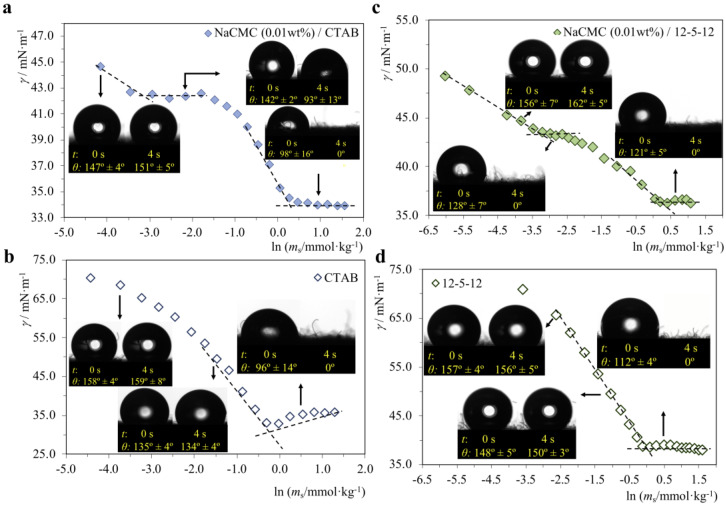
Contact angle (*θ*) measurements for water droplets on a hydrophobic substrate, performed in the surfactant concentrations indicated by the arrows: (**a**) NaCMC/CTAB system; (**b**) neat CTAB; (**c**) NaCMC/12-5-12 system; (**d**) neat 12-5-12. The polymer concentration is constant at 0.01 wt%. The *θ* values shown are for either 0 s or 4 s after droplet deposition.

**Figure 8:**
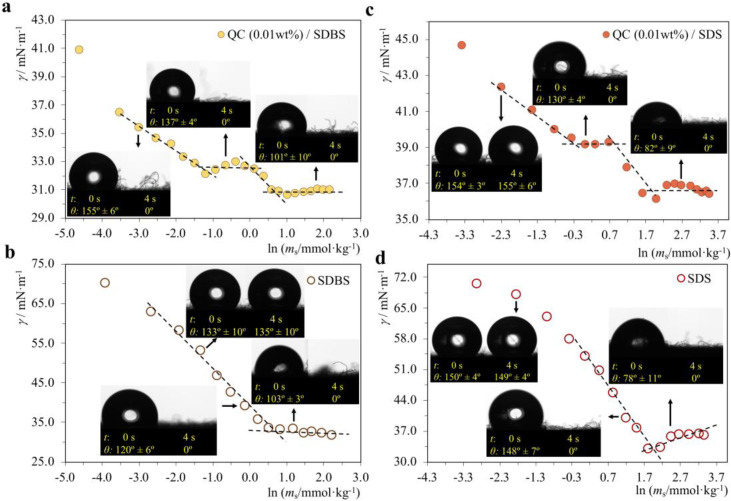
Contact angle (*θ*) measurements for water droplets on a hydrophobic substrate, performed in the surfactant concentrations indicated by the arrows: (**a**) QC/SDBS system; (**b**) neat SDBS; (**c**) QC/SDS system; (**d**) neat SDS. The polymer concentration is constant at 0.01 wt%. The *θ* values shown are for either 0 s or 4 s after droplet deposition.

NaCMC/CTAB, NaCMC/12-5-12, QC/SDBS and QC/SDBS mixtures were evaluated in concentrations along the surface tension curves (before *cac*, at the *cac* plateau, and after *cmc*^p^), and the results are shown in Figure 7 (P^−^/S^+^ systems) and Figure 8 (P^+^/S^−^ systems). In all systems, the polymer concentration was kept at 0.01%, and the surfactant concentration varied. For proper comparison, the neat surfactants were studied at the same concentrations but without polymer. All contact angles were measured in the first contact of the droplet with the substrate (0 s), as well as after 4 s, in order to obtain a broader analysis of the hydrophilic effect.

As a first general observation from Figure 7 and Figure 8, we can say that in all 4 systems, in the presence of polymer, in the *cac* plateau, there is already an increase in the hydrophilicity of the substrate caused by the P/S aggregates. The same does not happen when the surfactant is at the same concentration but without the polymer. Therefore, we conclude that P/S interactions in the mixture clearly favor hydrophilicity.

#### 2.2.1. P^−^/S^+^ Systems

Figure 7 shows that in both the NaCMC/CTAB and NaCMC/12-5-12, the presence of polymer reduces the contact angles for identical surfactant concentrations. This also means that higher hydrophilicity is attained at lower surfactant concentrations when the polymer is present in the solution. One of the most relevant observations is that in the NaCMC/12-5-12 system (Figure 7c), right after the *cac*, there is a dramatic increase in the hydrophilicity of the substrate caused by the mixture (for t = 4 s, *θ* = 0°, meaning that the drop is absorbed). This effect does not happen when 12-5-12 is at the same concentration but without NaCMC. Moreover, in the NaCMC/CTAB system, the effect appears only after *cmc*^p^, which is at a much higher surfactant concentration than for NaCMC/12-5-12, indicating that from the two P/S mixtures, the latter is much more effective in promoting hydrophilicity. In both mixtures, after *cmc*^p^, since there is a high excess of surfactant (in fact, free surfactant micelles), it is not surprising that the drops are completely absorbed within 4 s. This effect is also noticeable in the neat surfactant curves after the *cmc*. However, since in the presence of polymer, the hydrophilicity is higher at lower surfactant concentrations, in a real situation it would not be necessary to use such a high concentration of surfactants to favor hydrophilicity. In the case of gemini surfactants, we only tested 12-5-12 as this compound has an intermediate spacer and the highest *cac* and *cmc*^p^. Nevertheless, similar behavior is reasonably expected for mixtures of NaCMC with 12-2-12 and 12-10-12.

#### 2.2.2. P^+^/S^−^ Systems

In the P^+^/S^−^ systems, we intended to assess whether the presence of the cationic polymer could detrimentally affect the hydrophilicity typically provided by anionic surfactants in fabric softeners. When cationic amphiphiles are used in softeners, they actively provide the softening effect but may impair hydrophilicity; therefore, anionic surfactants are also often required in the formulations. Here, we wanted to evaluate whether it would be beneficial or not to include the cationic polymer in a softener formulation in terms of hydrophilicity. For this, the QC/SDBS and QC/SDS systems were studied (Figure 8), and significantly, a much more pronounced hydrophilic behavior was observed than in the NaCMC mixtures with cationic surfactants. In the presence of QC, not only the hydrophilicity of the anionic surfactant is not negatively affected, but the cationic polymer promotes it by increasing its level at much lower concentrations of surfactant.

This effect is particularly remarkable in the QC/SDBS mixture (Figure 8a), where the droplet is absorbed even before the *cac*, which does not happen in the absence of the polymer (Figure 8b). In this system, the only difference is in the surfactant since the SDBS has a benzene group attached to the anionic head. In the QC/SDS system (Figure 8c), we observed that, in the presence of the polymer, the droplet is absorbed right after the *cac*, whereas when the polymer is not present, the droplet was only absorbed after the *cmc* plateau (Figure 8d).

These results are very promising from an environmental point of view, as the use of a green polymer in the formulations would help to reduce the amount of surfactant needed. Moreover, the polymers used in this work originate from cellulose extracted from textile waste, which represents not only a route for valuing this waste from the textile sector but also the promotion of a circular economy approach, as the derivative is back into the value chain of the sector.

## 3. Materials and Methods

### 3.1. Synthesis of Sodium Carboxymethyl Cellulose (NaCMC)

Cellulose was extracted from post-consumer textiles wastes according to our previous report [41]. For the synthesis of carboxymethylcellulose, the 2-propanol ACS grade and chloroacetic acid (≥99.5%) used were purchased from VWR Chemicals (Radnor, PA, USA). The synthesis of NaCMC was adapted from Xiao et al. [56] and reported before [41]. Here, NaCMC was obtained with DS = 0.61 and M_W_ ≈ 2.67 × 10^4^ g·mol^−1^.

### 3.2. Synthesis of Cationic Quaternized Cellulose (QC)

QC was synthesized from the reaction of cellulose with glycidyltrimethylammonium chloride (CHPTAC) in an aqueous solution of sodium hydroxide with urea. A solution of 100 mL of NaOH-Urea-H_2_O (7-12-81%) was cooled, and 2 g of cellulose extracted from textile wastes was added. This mixture remained under intense agitation for 5 min before being subjected to freezing for 24 h. Thereafter, the mixture was warmed to room temperature, and then, under constant stirring, 21 mL of glycidyltrimethylammonium chloride (CHPTAC) was added slowly. The etherification reaction was left stirring for 24 h at room temperature. Then, the mixture was neutralized with HCl and the product was further submitted to dialysis (for 3 days) and lyophilization. The degree of substitution (DS) of QC was obtained by CHNS elemental analysis using a Truspec 630-200-200 analyzer. The DS of QC was calculated by nitrogen content, yielding a value of 0.68; the M_W_ is ≈2.68 × 10^4^ g·mol^−1^.

### 3.3. Surfactants

The following surfactants were purchased from Sigma-Aldrich (St. Louis, MO, USA): CTAB (>98%), SDS (>98.5%), SDBS (99%) and Triton X-100 (99.9%). The gemini surfactants 12-2-12, 12-5-12 and 12-10-12 were synthesized and purified by us as described by Menger et al. [53,57]. Before use, CTAB was recrystallized in acetone, and SDS was purified with diethyl ether and then recrystallized 3 times with ethanol.

### 3.4. Sample Preparation

Concentrated stock solutions of surfactant and polymers were prepared separately by rigorously dispersing the weighed quantities of each solid in ultrapure Milli-Q^®^ water at room temperature. Both solutions were placed in a rotating stirrer for 24 h at room temperature to allow complete homogenization. The surfactant/polymer samples were prepared by adding the polymer solutions to the surfactant solutions, followed by a rotating stirrer for 24 h at 25 °C to reach equilibrium. The surfactant concentration is expressed by molality and the polymer concentration in weight percentage.

### 3.5. Surface Tension Studies

For the surface tension studies of the neat surfactant and polymer-surfactant (P/S) systems, a DCAT11 tensiometer from Dataphysics GmbH (Filderstadt, Germany) with a Pt-Y alloy Wilhelmy plate was used. The temperature was kept constant by using a thermostated Julabo F20 circulating water bath (Seelbach, Germany) set to 25.0 °C for all systems except CTAB, set to 30.0 °C, due to its Krafft temperature (≈26–27 °C).

### 3.6. Zeta Potential and pH Studies

The zeta potential, *ζ*, of the P/S systems at several points of the tension surface curve was measured using a Zeta Sizer Nano ZS (Malvern, UK) and DTS 1060C disposable zeta cells from Malvern, at 25.0 °C. The electrophoretic mobility, μ, was measured using a combination of electrophoresis and laser Doppler velocimetry techniques, and the *ζ*-potential was calculated from μ using the Henry equation, with a dielectric constant of 78.5, a medium viscosity of 0.89 cP, and an *f*(*κa*) function value of 1.5 (Smoluchowsky approximation). The pH studies were carried out in an inoLAB^®^ pH 730 pH meter from WTW (Weilheim, Germany) duly calibrated at 25.0 °C. For both determinations, triplicate samples were measured 5 times each at 25.0 °C.

### 3.7. Contact Angle Measurements

To measure the static contact angle (CA), an Attension Theta Lite Optical Tensiometer from Biolin Scientific (Gothenburg, Sweden), with OneAttension software, was used. The measurements were performed with different polymer systems at several points of surface tension curves. The droplet volume was 5 μL. An average of 5–8 drops were made for each sample, and the contact angle was determined in 4 different frames at the first contact of the drop with the substrate and after 4 s. A hydrophobic textile was used as a substrate to verify the change in hydrophilicity caused by the different polymer-surfactant mixtures. The textile substrate was stretched on a support so that the roughness did not interfere with the measurement [57].

## 4. Conclusions

In this work, we studied the interactions, in aqueous solution, of two cellulose derivatives—NaCMC (anionic) and QC (cationic)—with surfactants that are commonly used in the textile industry, with the aim of evaluating the interfacial, aggregation and wetting properties of different polymer/surfactant (P/S) mixtures. These studies are not only fundamentally interesting but relevant in the context of the development of fabric softener and detergent formulations. From surface tension studies, we found that for oppositely charged systems (namely, NaCMC with CTAB or bis-quat gemini surfactants and QC with SDBS or SBS) electrostatic interactions lead to a strong association in solution. This is manifested essentially in two ways: in the form of a critical association concentration for the P/S mixtures (indicating the formation of P/S mixed aggregates) that is typically 15–20 times lower than the neat surfactant *cmc*; and in the enhanced surface activity of the P/S mixture compared to the polymer and surfactant individually. Interestingly, and counter to expectation, we also found a strong surface activity for a mixture of similarly charged polymer and surfactant, the QC/CTAB system. Regarding hydrophilicity, from contact angle measurements, we observed that the P/S mixtures lead to the enhanced hydrophilicity of a textile substrate at much lower surfactant concentrations than the surfactant alone (in particular in the QC/SDBS and QC/SDS systems). These results can be of great interest for the development of fabric softeners based on cationic cellulosic polymers, whereby the addition of small amounts of surfactant could improve hydrophilicity without potentially impairing the softening effect.

## Figures and Tables

**Table 1:** Interfacial parameters obtained from the surface tension curves for CTAB and NaCMC/CTAB mixtures and zeta potential and pH values of points marked by arrows in Figure 2b.

Interfacial Properties				
	*c*_NaCMC_/wt%			CTAB
	0.01	0.05	0.1	
*cac*/mmol·kg^−1^	0.038 ± 0.005	0.041 ± 0.007	0.035 ± 0.004	---
*cmc*^p^/mmol·kg^−1^	1.3 ± 0.1	3.0 ± 0.6	4.4 ± 0.9	---
*cmc*/mmol·kg^−1^	---	---	---	0.95 ± 0.04
*γ_cac_*/mN·m^−1^	41.8 ± 0.3	40.1 ± 0.2	39.7 ± 0.3	---
*γ_cmc_*^p^/mN·m^−1^	34.2 ± 0.2	34.0 ± 0.5	33.8 ± 0.2	---
*γ_cmc_*/mN·m^−1^	---	---	---	33.1 ± 0.2
**Zeta Potential and pH**				
***c*** **_NaCMC_** **/wt%**	***m*** **_CTAB_** **/mmol·kg^−1^**	***ζ* /mV**	**pH**	
0.01	0	−43 ± 1	7.4 ± 0.3	
	0.010	−49 ± 2	7.0 ± 0.1	
	0.10	−37 ± 1	7.0 ± 0.5	
	10	+35 ± 2	6.4 ± 0.3	
0.05	0	−46 ± 2	7.0 ± 0.2	
	0.010	−48 ± 1	6.8 ± 0.1	
	0.20	−40 ± 1	6.8 ± 0.2	
	10	+32 ± 1	6.6 ± 0.4	
0.1	0	−44 ± 2	7.5 ± 0.2	
	0.010	−50 ± 1	7.1 ± 0.2	
	0.20	−41 ± 1	7.2 ± 0.2	
	10	+34 ± 1	7.0 ± 0.4	
0	10	+42 ± 2	5.9 ± 0.1	

**Table 2:** Interfacial parameters obtained from the surface tension curves for 12-5-12 and NaCMC/12-5-12 mixtures and zeta potential and pH values of points marked by arrows in Figure 3b.

Interfacial Properties				
	*c*_NaCMC_/wt%			12-5-12
	0.01	0.05	0.1	
*cac*/mmol·kg^−1^	0.041 ± 0.001	0.061 ± 0.004	0.064 ± 0.009	---
*cmc*^p^/mmol·kg^−1^	1.30 ± 0.09	1.48 ± 0.07	2.65 ± 0.08	---
*cmc*/mmol·kg^−1^	---	---	---	0.87 ± 0.02
*γ_cac_*/mN·m^−1^	43.4 ± 0.6	40.6 ± 0.3	40.7 ± 0.1	---
*γ_cmc_*^p^/mN·m^−1^	36.2 ± 0.5	36.4 ± 0.9	36.0 ± 0.1	---
*γ_cmc_*/mN·m^−1^	---	---	---	38.4 ± 0.8
**Zeta Potential and pH**				
***c*_NaCMC_/wt%**	***m*_12-5-12_/mmol·kg^−1^**	***ζ*/mV**	**pH**	
0.01	0	−43 ± 1	7.4 ± 0.3	
	0.010	−48 ± 2	7.1 ± 0.5	
	0.080	−38 ± 1	6.6 ± 0.4	
	3.0	+25 ± 2	6.7 ± 0.1	
0.05	0	−46 ± 2	7.0 ± 0.2	
	0.010	−49 ± 1	7.2 ± 0.1	
	0.40	−43 ± 1	7.1 ± 0.1	
	8.0	+21 ± 1	6.8 ± 0.1	
0.1	0	−44 ± 2	7.5 ± 0.2	
	0.010	−49 ± 1	7.3 ± 0.1	
	0.40	−45 ± 1	7.2 ± 0.2	
	8.0	+19 ± 1	7.0 ± 0.1	
0	3.0	+57 ± 1	7.3 ± 0.5	

**Table 3:** Interfacial parameters obtained from the surface tension curves for 12-n-12 and NaCMC/12-n-12 mixtures (25.0 °C), and zeta potential and pH values of points marked by arrows in Figure 4b.

Interfacial parameters			
NaCMC (0.01 wt%)/12-n-12			
	12-2-12	12-5-12	12-10-12
*cac*/mmol·kg^−1^	0.018 ± 0.002	0.041 ± 0.001	0.025 ± 0.002
*cmc*^p^/mmol·kg^−1^	0.99 ± 0.04	1.30 ± 0.09	0.52 ± 0.06
*cmc*/mmol·kg^−1^	0.81 ± 0.02	0.87 ± 0.02	0.42 ± 0.01
*γ_cac_*/mN·m^−1^	42.1 ± 0.6	43.4 ± 0.6	40.9 ± 0.2
*γ_cmc_*^p^/mN·m^−1^	28.0 ± 1	36.2 ± 0.5	37.2 ±0.3
*γ_cmc_*/mN·m^−1^	30.9 ± 0.8	38.4 ± 0.8	40.8 ± 0.1
**Zeta Potential and pH**			
***c*_NaCMC_ = 0.01 wt%**			
**12-n-12**	***m*_12-n-12_/mmol·kg^−1^**	***ζ*/mV**	**pH**
12-2-12	0.010	−48 ± 1	7.1 ± 0.1
	0.060	−45 ± 1	6.9 ± 0.2
	3.0	+37 ± 2	7.0 ± 0.1
12-5-12	0.010	−48 ± 2	7.1 ± 0.5
	0.080	−38 ± 1	6.6 ± 0.4
	3.0	+25 ± 2	6.7 ± 0.1
12-10-12	0.010	−48 ± 2	7.3 ± 0.1
	0.080	−37 ± 1	7.1 ± 0.2
	3.0	+25 ± 2	7.4 ± 0.2
**neat systems**			
12-2-12	3.0	+52 ± 3	7.0 ± 0.2
12-5-12	3.0	+54 ± 2	7.1 ± 0.3
12-10-12	3.0	+57 ± 3	6.9 ± 0.5
NaCMC 0.01 wt%	---	−43 ± 1	7.4 ± 0.3

**Table 4:** Interfacial parameters obtained from the surface tension curves for SDBS and QC/SDBS mixtures (25.0 °C) and zeta potential and pH values of points marked by arrows in Figure 5b.

Interfacial properties				
	*c*_QC_/wt%			SDBS
	0.01	0.05	0.1	
*cac*/mmol·kg^−1^	0.33 ± 0.03	0.32 ± 0.04	0.35 ± 0.08	---
*cmc*^p^/mmol·kg^−1^	2.2 ± 0.4	3.0 ± 0.3	4.2 ± 0.2	---
*cmc*/mmol·kg^−1^	---	---	---	1.58 ± 0.06
*γ_cac_*/mN·m^−1^	32.2 ± 0.3	31.6 ± 0.6	31.6 ± 0.3	---
*γ_cmc_*^p^/mN·m^−1^	30.5 ± 0.2	29.0 ± 0.5	30.0 ± 0.2	---
*γ_cmc_*/mN·m^−1^	---	---	---	34.7 ± 0.5
**Zeta Potential and pH**				
***c*_CQ_/wt%**	***m*_SDBS_/mmol·kg^−1^**	***ζ*/mV**	**pH**	
0.01	0	+31 ± 2	6.5 ± 0.2	
	0.10	+26 ± 1	7.0 ± 0.3	
	0.50	+16 ± 1	6.9 ± 0.1	
	8.0	−37 ± 2	7.2 ± 0.1	
0.05	0	+36 ± 3	7.2 ± 0.2	
	0.10	+23 ± 2	7.3 ± 0.2	
	0.50	+10 ± 1	7.2 ± 0.2	
	8.0	−41 ± 1	7.5 ± 0.1	
0.1	0	+38 ± 2	8.4 ± 0.3	
	0.10	+22 ± 3	7.6 ± 0.3	
	0.50	+14 ± 1	7.7 ± 0.3	
	8.0	−39 ± 1	7.8 ± 0.1	
0	8.0	−56 ± 3	7.4 ± 0.1	

**Table 5:** Interfacial parameters obtained from the surface tension curves for CTAB (30.0 °C) and QC/CTAB mixtures (25.0 °C).

	*c*_QC_/wt%			CTAB
	0.01	0.1	0.5	
*cmc*_min_/mmol·kg^−1^	0.54 ± 0.03	0.23 ± 0.02	0.08 ± 0.01	---
*cmc*^p^/mmol·kg^−1^	1.2 ± 0.1	0.84 ± 0.09	0.43 ± 0.09	0.95 ± 0.04
*γ_cmc_*_min_/mN·m^−1^	32.1 ± 0.3	32.5 ± 0.4	32.2 ± 0.3	---
*γ_cmc_*^p^/mN·m^−1^	35.0 ± 0.2	33.9 ± 0.5	34.2 ± 0.3	33.1 ± 0.2

## Data Availability

Data are available in the article and in the Supplementary Materials.

## Supplementary Materials

The following supporting information can be downloaded at: https://www.mdpi.com/article/10.3390/molecules28083454/s1. Figure S1: Surface tension curves for neat SDS and QC/SDS mixtures (25.0 °C), where the polymer (P) concentration in each curve is constant and equal to 0.01, 0.05 or 0.1 wt%, and the surfactant (S) concentration is varied: a, all P/S systems, inset with SDS curve; b, individual P/S curves. Table S1: Interfacial parameters obtained from the surface tension curves for SDS and QC/SDS mixtures (25.0 °C) and zeta potential and pH values of points marked by arrows in Figure S1b. Figure S2: Surface tension curves for: a, neat SDBS and NaCMC/SDBS; b, neat SDS and NaCMC/SDS (25.0 °C), where the polymer (P) concentration in each curve is constant and equal to 0.01 wt%, and the surfactant (S) concentration is varied. Figure S3: Surface tension curves for neat TX-100, NaCMC/TX-100 and QC/TX-100 system (25.0 °C), where the polymer (P) concentration is constant and equal to 0.01 wt%, and the surfactant (S) concentration is varied.
