# Supplementary material for: Interactions between Ionic Cellulose Derivatives Recycled from Textile Wastes and Surfactants: Interfacial, Aggregation and Wettability Studies
Source: Molecules. 2023 Apr 13;28(8):3454. doi: 10.3390/molecules28083454 (PMC10144465; doi:10.3390/molecules28083454)
Supplement: Supplementary file 1 [file molecules-28-03454-s001.zip › molecules-2327756-supplementary.pdf]

molecules

Article

Interactions between ionic cellulose derivatives recycled from textile wastes and surfactants: interfacial, aggregation and wettability studies

Catarina Costa[1,2], André Viana[1,2], Isabel S. Oliveira[1] and Eduardo F. Marques[1,*]

1 CIQUP, IMS (Institute for Molecular Sciences), Departamento de Química e Bioquímica, Faculdade de Ciências, Universidade do Porto, Rua do Campo Alegre 1021, 4169-007 Porto, Portugal
2 CeNTI - Centre for Nanotechnology and Smart Materials, Rua Fernando Mesquita, 4760-034 Vila Nova de Famalicão, Portugal
* Correspondence: efmarque@fc.up.pt

Supplementary Materials

QC/SDS system – supplementary data

The *cac* of the QC/SDS is approx. 0.30 mmol·kg^{-1} and thus very similar to that of QC/SDBS. The decrease of the *cac* with respect to the *cmc* of SDS is by about 20 times and hence similar to the magnitude of decrease observed for the P$^-$/S$^+$ systems. Even though the extension of the *cac* plateau increases when the polymer concentration is increased from 0.05 to 0.1 wt%, it remains almost unchanged from 0.01 to 0.05 wt%; besides, we see that unlike in all other P/S systems studied here, the *cmc*^{p} practically does not change with polymer concentration remaining at about 6.4-6.6 mmol·kg^{-1} and, within error, similar to the *cmc* of the surfactant. This trend is thus somewhat out of line with what we observed so far. A possible explanation lies in the specific chemical properties of SDS: this surfactant is difficult to be obtained in a very pure form, due to contamination by dodecanol, a highly surface-active molecule (resulting from the autocatalytic degradation of the surfactant) that is hard to remove completely. Even small amounts of dodecanol (e.g. 0.1%) are enough to cause a dip in the surface tension curve near the *cmc* inflection point, thus effectively reducing (artificially) the determined *cmc*. This seems to be the case here (Figure 6a), as the measured *cmc* was 6.1 mmol·kg^{-1}, smaller than the *cmc* of a highly purified SDS, 8 mmol·kg^{-1} (we note, however, that the SDS batch used here was previously purified, as mentioned in section 2.1.3). As a consequence, the true *cmc*^{p} could be masked by the presence of dodecanol, and the usual trend mitigated. However, we did not proceed to further purifications of the surfactant, as the main goals of this study (P/S interactions) were anyway achieved. Concerning the variation of ζ (Table S1) and pH, the trends are very similar to those of the QC/SDBS system.

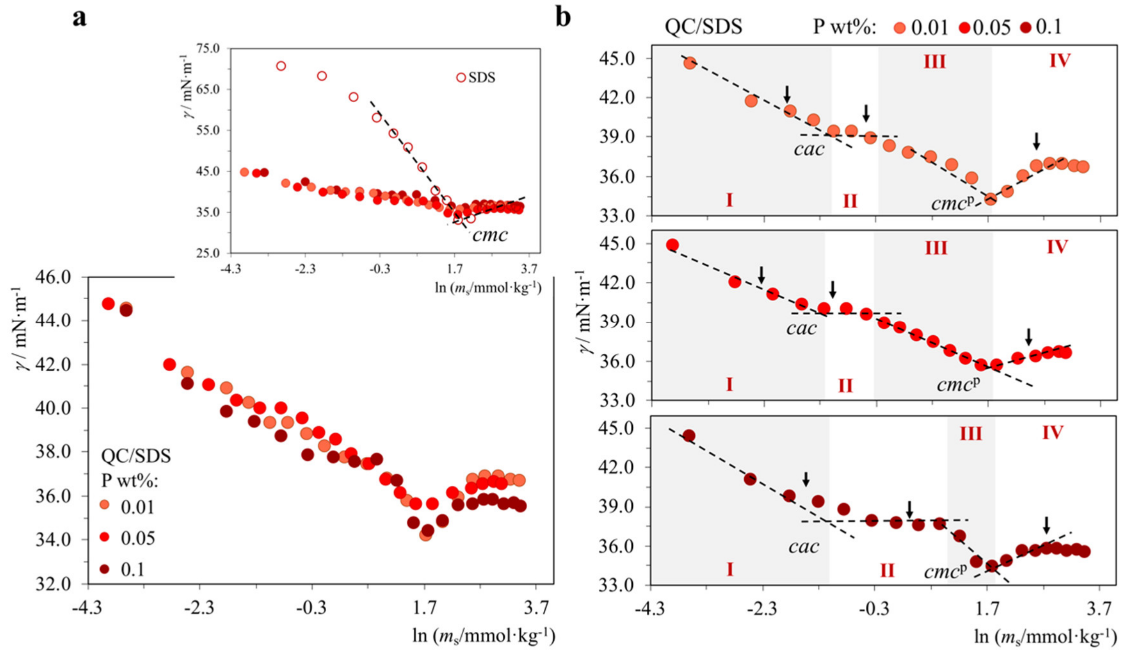

Figure S1. Surface tension curves for neat SDS and QC/SDS mixtures (25.0 °C), where the polymer (P) concentration in each curve is constant and equal to 0.01, 0.05 or 0.1 wt%, and the surfactant (S) concentration is varied: **a**, all P/S systems, inset with SDS curve, **b** individual P/S curves. Legend: *cmc*, neat surfactant critical micelle concentration; *cac*, critical association concentration, *cmc*^{p}, surfactant *cmc* in the presence of polymer.

Table S1. Interfacial parameters obtained from the surface tension curves for SDS and QC/SDS mixtures (25.0 °C) and zeta potential and pH values of points marked by arrows in Figure S1B.

Interfacial properties				
	c_{CQ} / wt%			SDS
	0.01	0.05	0.1	
cac /mmol·kg^{-1}	0.32 ± 0.08	0.21 ± 0.09	0.30 ± 0.09	---
cmc^{P} /mmol·kg^{-1}	6.6 ± 0.8	6.4 ± 0.5	6.3 ± 0.7	---
cmc /mmol·kg^{-1}	---	---	---	6.1 ± 0.5
γ_{cac} /mN·m^{-1}	39.5 ± 0.5	40.2 ± 0.3	38.0 ± 0.3	---
γ_{cmc^P} /mN·m^{-1}	35.0 ± 0.2	35.6 ± 0.5	36.8 ± 0.8	---
γ_{cmc} /mN·m^{-1}	---	---	---	33.9 ± 0.5
Zeta potential and pH				
c_{CQ} / wt%	m_{SDS} / mmol·kg^{-1}	ζ / mV	pH	
0.01	0	+31 ± 2	6.5 ± 0.2	
	0.10	+33 ± 1	6.9 ± 0.1	
	0.50	+16 ± 1	6.8 ± 0.2	
	12	–27 ± 1	6.7 ± 0.1	
0.05	0	+36 ± 3	7.2 ± 0.2	
	0.10	+29 ± 1	7.2 ± 0.1	
	0.50	+29 ± 1	6.9 ± 0.2	
	12	–26 ± 1	6.7 ± 0.4	
0.1	0	+38 ± 2	8.4 ± 0.3	
	0.10	+32 ± 2	7.2 ± 0.3	
	1.0	+29 ± 1	7.3 ± 0.1	
	12	–31 ± 1	7.8 ± 0.2	
0	12	–56 ± 3	6.0 ± 0.1	

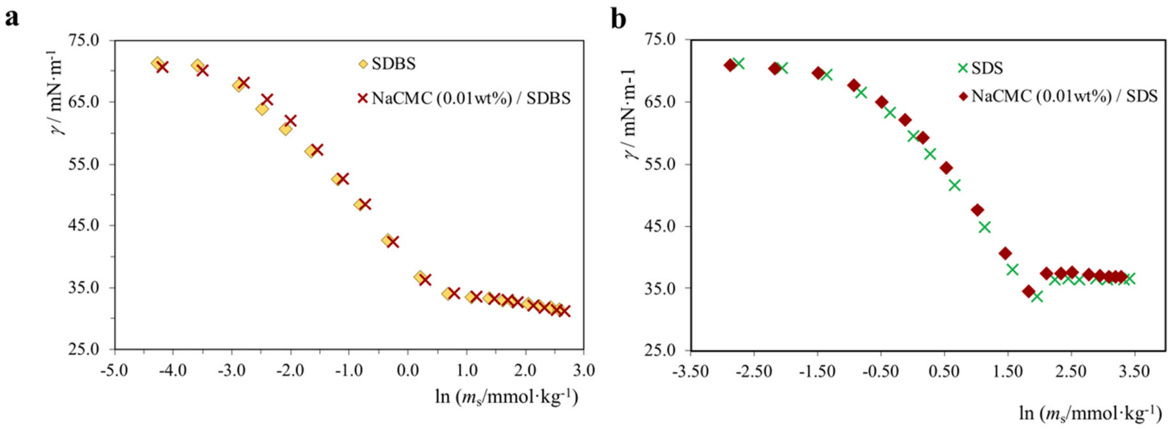

Figure S2. Surface tension curves for: **a**, neat SDBS and NaCMC/SDBS; **b**, neat SDS and NaCMC/SDS (25.0 °C), where the polymer (P) concentration in each curve is constant and equal to 0.01 wt%, and the surfactant (S) concentration is varied.

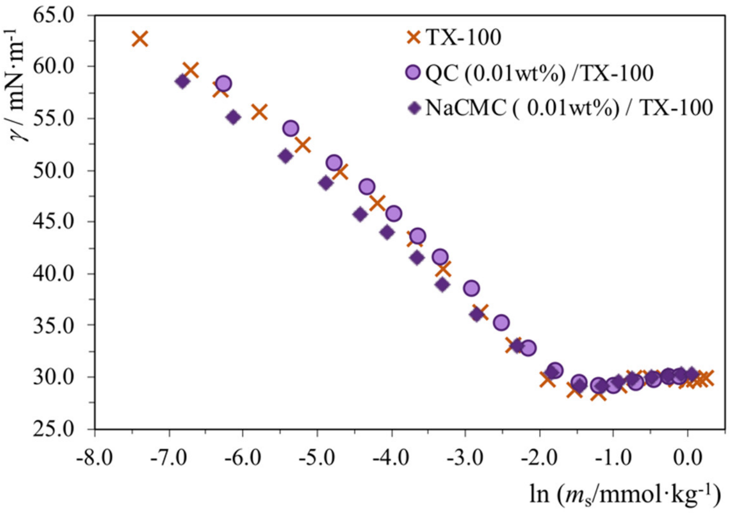

Figure S3. Surface tension curves for neat TX-100, NaCMC/TX-100 and QC/TX-100 system (25.0 °C), where the polymer (P) concentration is constant and equal to 0.01 wt%, and the surfactant (S) concentration is varied.
